# Far from Asymptopia: Unbiased High-Dimensional Inference Cannot Assume Unlimited Data

**DOI:** 10.3390/e25030434

**Published:** 2023-03-01

**Authors:** Michael C. Abbott, Benjamin B. Machta

**Affiliations:** Department of Physics, Yale University, New Haven, CT 06520, USA

**Keywords:** Bayesian inference, information geometry, posterior bias, model selection

## Abstract

Inference from limited data requires a notion of measure on parameter space, which is most explicit in the Bayesian framework as a prior distribution. Jeffreys prior is the best-known uninformative choice, the invariant volume element from information geometry, but we demonstrate here that this leads to enormous bias in typical high-dimensional models. This is because models found in science typically have an effective dimensionality of accessible behaviors much smaller than the number of microscopic parameters. Any measure which treats all of these parameters equally is far from uniform when projected onto the sub-space of relevant parameters, due to variations in the local co-volume of irrelevant directions. We present results on a principled choice of measure which avoids this issue and leads to unbiased posteriors by focusing on relevant parameters. This optimal prior depends on the quantity of data to be gathered, and approaches Jeffreys prior in the asymptotic limit. However, for typical models, this limit cannot be justified without an impossibly large increase in the quantity of data, exponential in the number of microscopic parameters.

## 1. Introduction

No experiment fixes a model’s parameters perfectly. Every approach to propagating the resulting uncertainty must, explicitly or implicitly, assume a measure of the space of possible parameter values. A badly chosen measure can introduce bias, and we argue here that avoiding such bias is equivalent to the very natural goal of assigning equal weight to each distinguishable outcome. However, this goal is seldom reached, either because no attempt is made, or because the problem is simplified by prematurely assuming the asymptotic limit of nearly infinite data. We demonstrate here that this assumption can lead to a large bias in what we infer from the parameters, in models with features typical of many-parameter mechanistic models found in science. We propose a score for such bias, and advocate for using a measure that makes this zero. Such a measure allows for unbiased inference without the need to first simplify the model to just the right degree of complexity. Instead, weight is automatically spread according to a lower effective dimensionality, ignoring details irrelevant to visible outcomes.

We consider models which predict a probability distribution p(x|θ) for observing data *x* given parameters θ. The degree of overlap between two such distributions indicates how difficult it is to distinguish the two parameter points, which gives a notion of distance on parameter space. The simplifying idea of information geometry is to focus on infinitesimally close parameter points, for which there is a natural Riemannian metric, the Fisher information [1,2]. This may be thought of as having units of standard deviations, so that along a line of integrated length *L* there are about *L* distinguishable points, and thus any parameter which can be measured to a few digits of precision has length L>100. It is a striking empirical feature of models in science that most have a few such long (or relevant) parameter directions, followed by many more short (or irrelevant) orthogonal directions [3,4,5,6]. The irrelevant lengths, all L<1, show a characteristic spectrum of being roughly evenly spaced on a log scale, often over many decades. As a result, much of the geometry of this Riemannian model manifold consists of features much smaller than 1, far too small to observe. However, the natural intrinsic volume measure, which follows from the Fisher metric, is sensitive to all of these unobservable dimensions, and as we demonstrate here, they cause this measure to introduce enormous bias.

**Figure 1:**
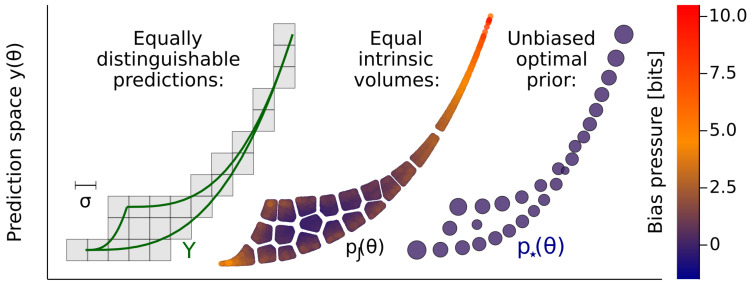
The natural volume is a biased measure for the space of distinguishable outcomes. The left panel outlines the space of possible predictions *Y*; the observed *x* is deterministic y(θ) plus measurement noise. With the scale of the noise σ as shown, the upper half is effectively one-dimensional. The center panel shows a sample from the volume measure $p_{J}\left(\theta \right)$, divided into blocks of equal weight. These are strongly influenced by the unobservable thickness of the upper portion. Points are colored by bias pressure b(θ), which we define in Equation (5). The right panel shows the explicitly unbiased optimal measure $p_{⋆}\left(\theta \right)$, which gradually adjusts from two- to one-dimensional behavior (The model is Equation (6) with $a_{1}=0.8$, $a_{2}=0.2$, and $k_{1}\geq k_{2}$, observed at times t=1,3 each with Gaussian noise σ=0.1).

To avoid this problem, we need a measure tied to the Fisher length scale L≈1, instead of one from the continuum. Locally, this length scale partitions dimensions into relevant and irrelevant, which in turn approximately factorizes the volume element into a relevant part and what we term the *irrelevant co-volume*. The wild variations of this co-volume are the source of the bias we describe, and it is rational to ignore them. As we illustrate in Figure 1 for a simple two-parameter model, equally distinguishable predictions do not correspond to equal intrinsic volumes, and this failure is detected by a score we call *bias pressure*. The measure $p_{⋆}\left(\theta \right)$ for which this score is everywhere zero, by contrast, captures relevant distinguishability and ignores the very thin irrelevant direction. The same measure is also obtained by maximizing the information learned about parameter θ from seeing data *x* [7,8,9], or equivalently from a particular minimax game [10,11,12]. Since $p_{⋆}\left(\theta \right)$ is usually discrete [9,13,14,15,16,17,18], it can be seen as implementing a length cutoff, replacing the smooth differential-geometric view of the model manifold with something quantized [19].

In the Bayesian framework, the natural continuous volume measure $p_{J}\left(\theta \right)$ is known as Jeffreys prior, and is the canonical example of an uninformative prior: a principled, ostensibly neutral choice. It was first derived based on invariance considerations [20], and can also be justified by information- or game-theoretic ideas, provided these are applied in the limit of infinitely many repetitions [7,8,17,21,22]. This asymptotic limit often looks like a technical trick to simplify derivations. However, in realistic models, this limit is very far from being justified, exponentially far in the number of parameters, often requiring an experiment to be repeated for longer than the age of the universe. We demonstrate here that using the prior derived in this limit introduces a large bias in such models. Furthermore, we argue that such bias, and not only computational difficulties, has prevented the wide use of uninformative priors.

The promise of principled ways of tracking uncertainty, Bayesian or otherwise, is to free us from the need to select a model with precisely the right degree of complexity. This idea is often encountered in the context of overfitting, where the maximum likelihood point of an overly complex model gives worse predictions. The bias discussed here is a distinct way for overly complex models to give bad predictions. We begin with toy models in which the number of parameters can be easily adjusted. However, in the real models of interest, we cannot trivially tune the number of parameters. This is why we wish to find principled methods which are not fooled by the presence of many irrelevant parameters.

## 2. Results

We consider a model to be characterized by the likelihood p(x|θ) of observing data x∈X when the parameters are θ∈Θ. In such a model, the Fisher information metric (FIM) measures the distinguishability of nearby points in parameter space as a distance $ds^{2}\left(\theta \right)=\sum_{\mu ,\nu =1}^{d}g_{\mu \nu }\left(\theta \right)d\theta^{\mu }d\theta^{\nu }$, where

(1) $$g_{\mu \nu }\left(\theta \right)=-\int \;dx\;p\left(x|\theta \right)\;\partial_{\mu }\partial_{\nu }\log p\left(x|\theta \right).$$

For definiteness, we may take points separated along a geodesic by a distance $L=\int \sqrt{ds^{2}\left(\theta \right)}>1$ to be distinguishable. Intuitively, though incorrectly, the *d*-dimensional volume implied by the FIM might be thought to correspond to the total number of distinguishable parameter values inferable from an experiment:

$$Z=\int \;d\theta \sqrt{\det g(\theta )}.$$

However, this counting makes a subtle assumption that all structure in the model has a scale much larger than 1. When many dimensions are smaller than 1, their lengths weigh the effective volume along the larger dimensions, despite having no influence on distinguishability.

The same effect applies to the normalized measure, Jeffreys prior:

(2) $$p_{J}\left(\theta \right)=\frac{1}{Z}\sqrt{\det g(\theta )}.$$

This measure’s dependence on the *irrelevant co-volume* is an under-appreciated source of bias in posteriors derived from this prior. The effect is most clearly seen when the FIM is block-diagonal, $g=g_{\mathrm{rel}}⊕g_{\mathrm{irrel}}$. Then the volume form factorizes exactly, and the relevant effective measure is the $\sqrt{\det g_{\mathrm{rel}}\left(\theta_{\mathrm{rel}}\right)}$ factor times $V_{\mathrm{irrel}}\left(\theta_{\mathrm{rel}}\right)$, an integral over the irrelevant dimensions.

A more principled measure of the (log of the) number of distinguishable outcomes is the mutual information between parameters and data, I(X;Θ):

$$I\left(X;\Theta \right)=\int \;d\theta \;p\left(\theta \right)D_{\mathrm{KL}}\left[p(x|\theta )∥p(x)\right]$$

where $D_{\mathrm{KL}}$ is the Kullback–Leibler divergence between two probability distributions, which are not necessarily close: p(x)=∫dθp(θ)p(x|θ) is typically much broader than p(x|θ). Unlike the volume *Z*, the mutual information depends on the prior p(θ). Past work, both by ourselves and others, has advocated for using the prior, which maximizes this mutual information, with [8] or without [9] taking the asymptotic limit:

(3) $$p_{⋆}\left(\theta \right)=\underset{p(\theta )}{\mathrm{argmax}}I\left(X;\Theta \right).$$

The same prior arises from a minimax game in which you choose a prior, your opponent chooses the true θ, and you lose the (large) KL divergence [10,11,12]:

(4) $$p_{⋆}\left(\theta \right)=\underset{p(\theta )}{\mathrm{argmin}}\max_{\theta }D_{\mathrm{KL}}\left[p(x|\theta )∥p(x)\right].$$

Here we stress a third perspective, defining a quantity we call *bias pressure* which captures how strongly the prior disfavors predictions from a given point:

(5) $$b\left(\theta \right)=\frac{\partial I(X;\Theta )}{\partial p(\theta )}{|}_{\int \;d\theta \;p(\theta )=1}=D_{\mathrm{KL}}\left[p(x|\theta )∥p(x)\right]-I\left(X;\Theta \right).$$

The optimal $p_{⋆}\left(\theta \right)$ has b(θ)=0 on its support, and can be found by minimizing $B=\max_{\theta }b\left(\theta \right).$ Other priors have b(θ)>0 at some points, indicating that I(X;Θ) can be increased by moving weight there (and away from points where b(θ)<0). We demonstrate below that b(θ) deserves to be called a bias, as it relates to large deviations of the posterior center of mass. We enact this by presenting a number of toy models, chosen to have information geometry similar to that typically found in mechanistic models from many scientific fields [5].

### 2.1. Exponential Decay Models

The first model we study involves inferring rates of exponential decay. This may be motivated, for instance, by the problem of determining the composition of a radioactive source containing elements with different half-lives, using Geiger counter readings taken over some period of time. The mean count rate at time *t* is

(6) $$y_{t}\left(\theta \right)=\sum_{\mu =1}^{d}a_{\mu }e^{-k_{\mu }t},\;k_{\mu }=e^{-\theta_{\mu }}>0.$$

We take the decay rates as parameters, and fix the proportions $a_{\mu }$, usually to $a_{\mu }=1/d$, thus initial condition $y_{0}=1$. If we make observations at *m* distinct times *t*, then the prediction *y* is an *m*-vector, restricted to a compact region $Y\subset {\left[0,1\right]}^{m}$. For radioactivity, we would expect to observe $y_{t}$ plus Poisson noise, but the qualitative features are the same if we simplify to Gaussian noise with constant width σ:

(7) $$p\left(x|\theta \right)=e^{-{\left|x-y(\theta )\right|}^{2}/2\sigma^{2}}/{\left(2\pi \sigma^{2}\right)}^{m/2}.$$

The Fisher metric then simplifies to be the Euclidean metric in the space of predictions *Y*, pulled back to parameter space Θ:

$$g_{\mu \nu }\left(\theta \right)=\frac{1}{\sigma^{2}}\sum_{t,t^{\prime }}^{m}\frac{\partial y_{t}}{\partial \theta^{\mu }}\frac{\partial y_{t^{\prime }}}{\partial \theta^{\nu }}\;\delta_{tt^{\prime }}$$

thus, plots of p(y) in $R^{m}$ will show Fisher distances accurately. This model is known to be ill conditioned, with many small manifold widths and many small FIM eigenvalues when *d* is large [23].

With just two dimensions, d=m=2, Figure 1 shows the region $Y\subset R^{2}$, Jeffreys prior $p_{J}\left(\theta \right)$, and the optimal prior $p_{⋆}\left(\theta \right)$, projected to densities on *Y*. Jeffreys is uniform $p_{J}\left(y\right)\propto 1$ (since the metric is constant in *y*), and hence always weights a two-dimensional area, both where this is appropriate and where it’s not. The upper portion of *Y* in the figure is thin compared to σ, so the points we can distinguish are those separated vertically: the model is effectively one-dimension there. Jeffreys does not handle this well, which we illustrate in two ways. First, the prior is drawn divided into 20 segments of equal weight (equal area), which roughly correspond to distinguishable differences where the model is two-dimensional, but not where it becomes one-dimensional. Second, the points are colored by b(θ), which detects this effect, and gives large values at the top (about 10 bits). The optimal prior avoids these flaws by smoothly adjusting from the one- to the two-dimensional part of the model [9].

The claim that some parts of the model are effectively one-dimensional depends on the amount of data gathered. Independent repetitions of the experiment have overall likelihood $p\left(x^{M}|\theta \right)=\prod_{i=1}^{M}p\left(x^{\left(i\right)}|\theta \right)$, which will always scale the FIM by *M*, hence all distances by a factor $\sqrt{M}$. This scaling is exactly equivalent to smaller Gaussian noise σ. Increasing *M* increases the number of distinguishable points, and large enough *M* (or small enough σ) can eventually make any nonzero length larger than 1. Thus, the amount of data gathered affects which parameters are relevant. However, notice that such repetition has no effect at all on $p_{J}\left(\theta \right)$, since the scale of $g_{\mu \nu }\left(\theta \right)$ in Equation (2) is canceled by *Z*. In this sense, it is already clear that Jeffreys prior belongs to the fixed point of repetition, i.e., to the asymptotic limit M→∞.

**Figure 2:**
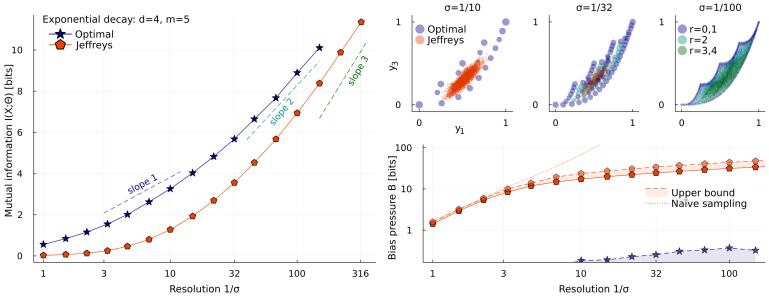
The effect of varying the noise level σ on a fixed model. The model is (6) with d=4 parameters, observed at m=5 times t=1,2,…5. Top right, the optimal prior $p_{⋆}\left(\theta \right)$ has all of its weight on 0- and 1-dimensional edges at large σ, but adjusts to fill in the bulk at small σ (colors indicate the dimension *r* of the 4-dimensional shape’s edge on which a point is located, the rank of the FIM there). Jeffreys prior $p_{J}\left(\theta \right)$ is independent of σ, and has nonzero density everywhere, but a sample of $10^{6}$ points is largely located near the middle of the shape. Left, the slope of $I_{⋆}\left(X;\Theta \right)∼d_{\mathrm{eff}}\log1/\sigma$ gives a notion of effective dimensionality; in the asymptotic limit σ→0, we expect $d_{\mathrm{eff}}=d=4$. Bottom right, the worst-case bias pressure $B=\max_{\theta }b\left(\theta \right)$ is always zero for $p_{⋆}\left(\theta \right)$, up to the numerical error, but remains nonzero for $p_{J}\left(\theta \right)$ even in the asymptotic limit. The Appendix A describes how upper and lower bounds for *B* are calculated.

Figure 2 shows a more complicated version of the model (6), with d=4 parameters, and looks at the effect of varying the noise level σ. Jeffreys prior always fills the 4-dimensional bulk, but at moderate σ, most of the distinguishable outcomes are located far from this mass. At large σ, equivalent to few repetitions, all the weight of the optimal prior is on zero- and one-dimensional edges. As more data are gathered, it gradually fills in the bulk, until, in the asymptotic limit σ→0, it approaches Jeffreys prior [12,17,21,24]. However, while $p_{⋆}\left(\theta \right)$ approaches a continuum at any interior point [25], it remains discrete at Fisher distances ∼1 from the boundary. The worst-case bias pressure detects this; hence, the maximum for Jeffreys prior does not approach that for the optimal prior: BJ→0. However, since mutual information is dominated by the interior in this limit, we expect the values for $p_{J}\left(\theta \right)$ and $p_{⋆}\left(\theta \right)$ to agree in the limit: $I_{J}-I_{⋆}\to 0$.

One way to quantify the effective dimensionality is to look at the rate of increase in mutual information under repetition, or decreasing noise σ. Along a dimension with Fisher length L≫1, the number of distinguishable points is proportional to *L*, and thus a cube with $d_{\mathrm{eff}}$ large dimensions will have $\propto L^{d_{\mathrm{eff}}}$ such points. This motivates defining $d_{\mathrm{eff}}$ by

(8) $$I_{⋆}\left(X;\Theta \right)∼d_{\mathrm{eff}}\log L,\;L=\int \;\sqrt{ds^{2}}\propto 1/\sigma .$$

Figure 2 shows lines for slope $d_{\mathrm{eff}}=1,2,3$, and we expect $d_{\mathrm{eff}}\to d$ in the limit σ→0.

### 2.2. The Costs of High Dimensionality

**Figure 3:**
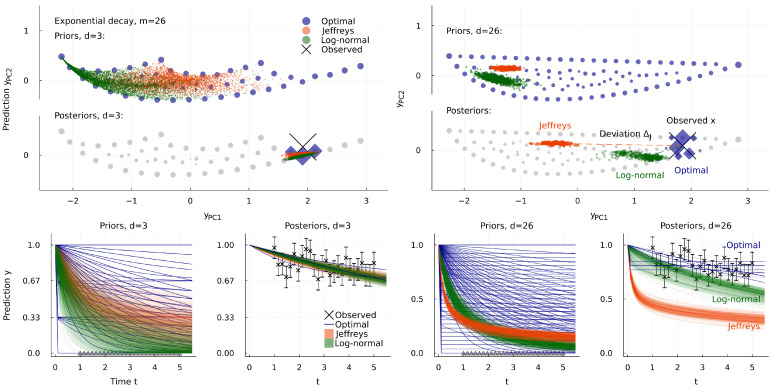
The effect of changing model dimension for fixed data and noise level. Left half, the exponential decay model of Equation (6) with d=3 parameters, observed with noise σ=0.1 at m=26 times in 1≤t≤5. Three priors are shown, drawn above by projecting onto the first two principal components of vector *y*, and below-left as a time course $y_{t}$ (each point on the upper plot is a line on the lower one). The corresponding posteriors are shown for a particular fixed *x*, which is the large cross in the upper plot (where the prior is shown again in light gray as a visual guide) and the series of points in the lower plot. In the d=3 model, all three posteriors are reasonable fits to the data. Right half, the similar model with d=26 parameters, for the same observations with the same noise. Here Jeffreys prior is much more strongly concentrated, favoring the part of the manifold where the irrelevant dimensions are largest. This has the effect of biasing the posterior far from the data, more than 20 standard deviations away. Figure 4 and Figure 5 explore the same setup further, including intermediate dimensions *d*. The log-normal prior is introduced in Equation (10).

**Figure 4:**
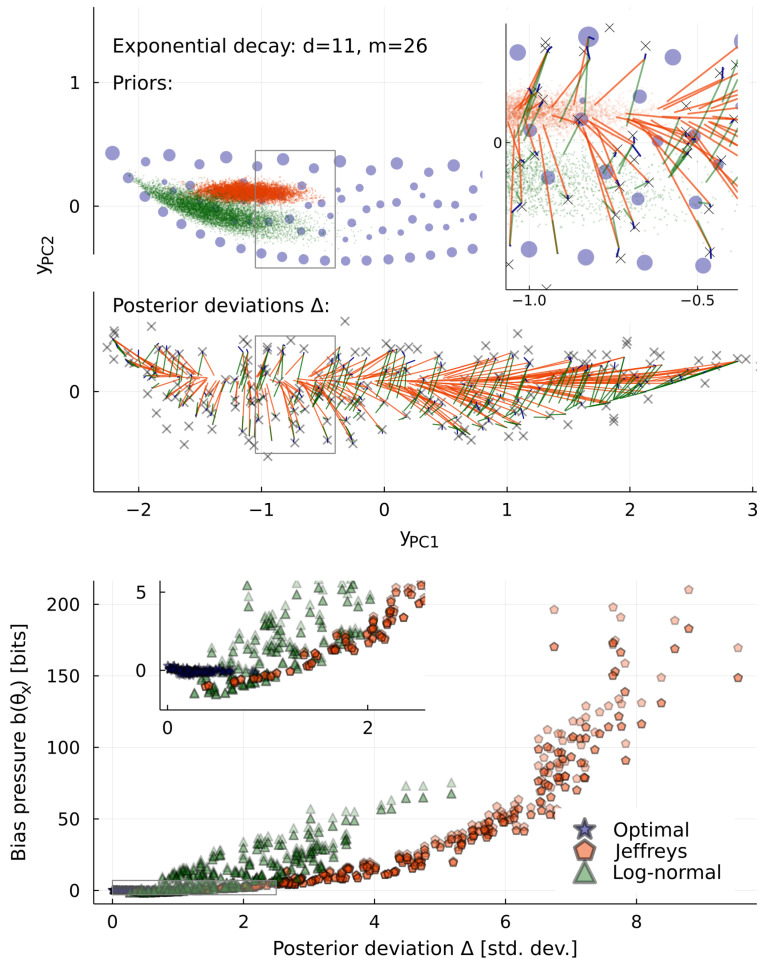
Posterior bias due to concentration of measure. Above, priors for the d=11 case of the model in Figure 3. We calculate the posterior for each at 100 points *x* (marked), and draw a line connecting the maximum likelihood point $y({\hat{\theta }}_{x})$ to the posterior center of mass ${\langle y(\theta )\rangle }_{x}$. Inset enlarges to show that there are blue lines too, for the optimal prior, most much shorter than the spacing of its atoms. Below, we compare the length of such lines (divided by σ=0.1) to the bias pressure $b({\hat{\theta }}_{x})$. Notice that b(θ) is sometimes negative (it has zero expectation value: ∫dθp(θ)b(θ)=0), although the worst-case $B=\max_{\theta }b\left(\theta \right)$ is non-negative. Each pair of darker and lighter points are a lower and an upper bound, explained in the Appendix A.

**Figure 5:**
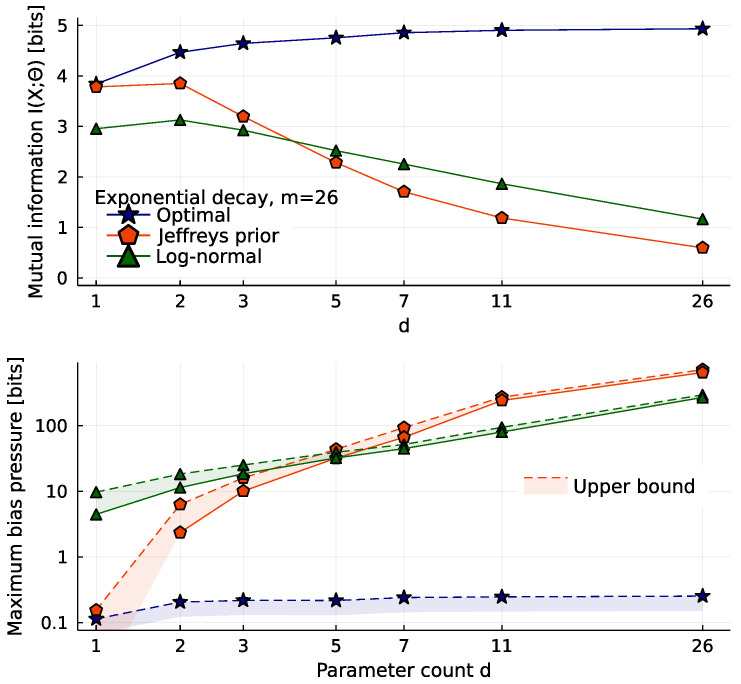
Information-theoretic scores for priors, as a function of dimensionality *d*. Like Figure 3, these models all describe the same data, with the same noise. Above, mutual information I(X;Θ)/log2 (all plots are scaled thus to have units of bits). The optimal prior ignores the addition of more irrelevant parameters, but Jeffreys prior is badly affected, and ends up capturing less than 1 bit. Below, worst-case bias pressure $\max_{\theta }b\left(\theta \right)/\log2$. This should be zero for the optimal prior, but our numerical solution has small errors. For the other priors, we plot lower and upper bounds, calculated using Bennett’s method [26], as described in the Appendix A. The bias of Jeffreys prior increases strongly with the increasing concentration of its weight in higher dimensions.

The problems of uneven measure grow more severe with more dimensions. To explore this, Figure 3, Figure 4 and Figure 5 show a sequence of models with 1 to 26 parameters. All describe the same data: observations at the same list of m=26 times in 1≤t≤5 with the same noise σ=0.1. While Jeffreys prior is nonzero everywhere, its weight is concentrated where the many irrelevant dimensions are largest. With a Monte Carlo sample of a million points, all are found within the small orange area on the right of Figure 3. For a particular observation *x*, we plot also the posterior p(θ|x) for each prior. The extreme concentration of weight in $p_{J}\left(\theta \right)$ in d=26 pulls this some 20 standard deviations away from the maximum likelihood point $y({\hat{\theta }}_{x})$. We call this distance the posterior deviation Δ; it is the most literal kind of bias in results.

Figure 4 compares the posterior deviation Δ to the bias pressure b(θ) defined in Equation (5). For each of many observations *x*, we find the maximum likelihood point ${\hat{\theta }}_{x}={\mathrm{argmax}}_{\theta }p\left(x|\theta \right)$, and calculate the distance from this point to the posterior expectation value of *y*:

(9) $$\Delta \left(x\right)=\frac{1}{\sigma }|y\left({\hat{\theta }}_{x}\right)-\int \;d\theta \;p\left(\theta |x\right)\;y\left(\theta \right)|.$$

Then, using the same prior, we evaluate the corresponding bias pressure, $b({\hat{\theta }}_{x})$. The figure shows 100 observations *x* drawn from $p_{⋆}\left(x\right)=\int d\theta \;p_{⋆}\left(\theta \right)\;p\left(x|\theta \right)$, and we believe this justifies the use of the word “bias” to describe b(θ). The figure is for d=11, but a similar relationship is seen in other dimensionalities.

Instead of looking at particular observations *x*, Figure 5 shows global criteria I(X;Θ) and $B=\max_{\theta }b\left(\theta \right)$. The optimal prior is largely unaffected by the addition of many irrelevant dimensions. Once d>3, it captures essentially the same information in any higher dimension and has zero bias (or near-zero bias, in our numerical approximation). We may think of this as a new invariance principle, that predictions should be independent of unobservable model details. This replaces one of the invariances of Jeffreys, that repetition of the experiment does not change the prior. Repetition invariance guarantees poor performance when we are far from the asymptotic limit, as we see here from the rapidly declining performance of Jeffreys prior with increasing dimension, capturing less than one bit in d=26. This decline in information is mirrored by a rise in the worst-case bias *B*.

Figure 3, Figure 4 and Figure 5 also show a third prior, which is log-normal in each decay rate $k_{\mu }=e^{\theta_{\mu }}>0$, that is, normal in terms of $\theta \in R^{d}$:

(10) $$p_{\mathrm{LN}}\left(\theta \right)\propto \prod_{\mu =1}^{d}e^{-{\left(\theta_{\mu }-\bar{\theta }\right)}^{2}/2{\bar{\sigma }}^{2}},\;\bar{\theta }=0,\;\bar{\sigma }=1\;\forall \mu .$$

This is not a strongly principled choice, but something like this is commonly used for parameters known to be positive. Here it produces better results than Jeffreys prior in high dimensions. We observe that it also suffers a decline in performance with increasing *d*, despite making no attempt to deliberately adapt to the high-dimensional geometry. The details of how well it works will, of course, depend on the values chosen for $\bar{\theta },\bar{\sigma }$, and more complicated priors of this sort can be invented. With enough free “meta-parameters” such as $\bar{\theta },\bar{\sigma }$, we can surely adjust such a prior to approximate the optimal prior, and in practice, such a variational approach might be more useful than solving for the optimal prior directly. We believe that worst-case bias $B=\max_{\theta }b\left(\theta \right)$ is a good score for this purpose, partly because its zero point is meaningful.

### 2.3. Inequivalent Parameters

Compared to these toy models, more realistic models often still have many parameter combinations poorly fixed by data, but seldom come in families that allow us to easily tune the number of dimensions. Instead of having many interchangeable parameters, each will often describe a different microscopic effect that we know to exist, even if we are not sure which combination of them will matter in a given regime [27]. To illustrate this, we now examine some models of enzyme kinetics, starting with the famous reaction:

(11) $$E+S\overset{k_{f}}{\underset{k_{r}}{⇌}}ES\overset{k_{p}}{\to }E+P$$

This summarises differential equations for the concentrations, such as $\partial_{t}\left[P\right]=k_{p}\left[ES\right]$ for the final product *P*, and $\partial_{t}\left[E\right]=-k_{f}\left[E\right]\left[S\right]+k_{r}\left[ES\right]+k_{p}\left[ES\right]$ for the enzyme, which combines with the substrate to form a bound complex.

**Figure 6:**
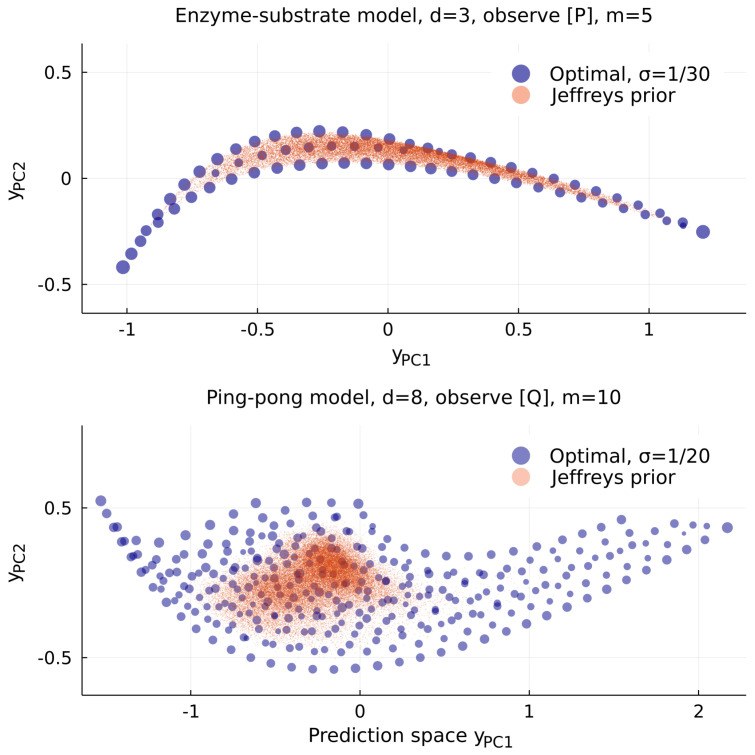
Priors for two models of enzyme kinetics. Above, the 3-parameter model from Equation (11), observing only the concentration of product [P] at times t=1,2,…5. Below, the 8-parameter model from Equation (12), observing only the final product [Q] at times t=1,2,…10. Here Jeffreys prior has worst-case bias B≈28 bits, comparable to the models in Figure 5 at similar dimension, while the optimal prior for the d=3 model has its weight on well-known 2-parameter approximations, including that of Michaelis and Menten, the edge structure of the d=8 model is much more complicated (for suitable initial conditions, it will include the d=3 model as an edge).

If the concentration of product [P] is observed at some number times, with some noise, and starting from fixed initial conditions, then this model is not unlike the toy model above. Figure 6 shows the resulting priors for the rate constants appearing in Equation (11). The shape of the model manifold is similar, and the optimal prior again places most of its weight along two one-dimensional edges, while Jeffreys prior places it in the bulk, favoring the region where all three rate constants come closest to having independently visible effects on the data. However, the resulting bias is not extreme in three dimensions.

The edges of this model are known approximations, in which certain rate constants become infinite (or equal), which we discuss in the Appendix A [28]. These approximations are useful in practice since each spans the full length of the most relevant parameter. However, the more difficult situation is when many different processes of comparable speed are unavoidably involved. The model manifold may still have many short directions, but the simpler description selected by $p_{⋆}\left(\theta \right)$ will tend to have weight on many different processes. In other words, the simpler model, according to information theory, is not necessarily one simpler model obtained by taking a limit, but instead, a mixture of many different analytic limits.

To see this, we consider a slightly more complicated enzyme kinetics model, the ping-pong mechanism with d=8 rate constants:

(12) $$\begin{array}{cc} E+A\to EA⇌E^{*}P\to E^{*}+P\\ E^{*}+B\to E^{*}B & ⇌EQ\to E+Q. \end{array}$$

Here $E^{*}$ is a deformed version of the enzyme *E*, which is produced in the reaction from *A* to *P*, and reverted in the reaction from *B* to final product *Q*. There are clearly many more possible limits in which some combination of the rate constants become large or small. Figure 6 shows that the optimal prior has weight on at least five different 1-edges, none of which is a good description by itself.

The concentration of weight seen in Jeffreys prior for these enzyme models is comparable to what we had before, with worst-case bias pressure B≈14 bits in d=3 and 28 bits in d=8. These examples share geometric features with many real models in science [5], and thus we believe the problems described here are generic.

## 3. Discussion

Before fitting a model to data, there is often a selection step to choose a model which is complex enough to fit the true pattern, but not so complex as to fit the noise. The motivation for this is clear in maximum likelihood estimation, where only one ${\hat{\theta }}_{x}$ is kept, and there are various criteria for making the trade-off [29,30,31]. The motivation is less clear in Bayesian analysis, where slightly different criteria can be derived by approximating p(x) [32,33]. We might hope that if many different points θ are consistent with the noisy data *x*, then the posterior p(θ|x) should simply have weight on all of them, encoding our uncertainty about θ.

Why, then, is model selection needed at all in Bayesian inference? Our answer here is that this is performed to avoid measure-induced bias, not overfitting. When using a sub-optimal prior, models with too much complexity do indeed perform badly. This problem is seen in Figure 5, in the rapid decline of scores I(X;Θ) or *B* with increasing *d*, and would also be seen in the more traditional model evidence p(x)—all of these scores prefer models with d≤3. However, the problem is not overfitting, since the extra parameters being added are irrelevant, i.e., they can have very little effect on the predictions $y_{t}\left(\theta \right)$. Instead, the problem is concentration of measure. In models with tens of parameters, this effect can be enormous: It leads to posterior expectation values Δ>20 standard deviations away from ideal, for the d=26 model with Jeffreys prior, and mutual information I<1 bit learned, and B>500 bits of bias. This problem is completely avoided by the optimal prior $p_{⋆}\left(\theta \right)$, which suffers no decline in performance with increasing parameter count *d*.

Geometrically, we can view traditional model selection as adjusting *d* to ensure that the model manifold only has dimensions of length L>1. This ensures that most of the posterior weight is in the interior of the manifold; hence, ignoring model edges is justified. By contrast, when there are dimensions of length L<1, the optimal posterior will usually have its weight at their extreme values, on several manifold edges, which are themselves simpler models [9]. Fisher lengths *L* depend on the quantity of data to be gathered, and repeating an experiment *M* times enlarges all by a factor $\sqrt{M}$. Large enough *M* can eventually make any dimension larger than 1, and thus repetition alters what *d* traditional model selection prefers. Similarly, repetition alters the effective dimensionality of $p_{⋆}\left(\theta \right)$. Some earlier work on model geometry studies a series in 1/M [22,33,34]; this expansion around L=∞ captures some features beyond the volume but is not suitable for models with dimensions L≪1.

Real models in science typically have many irrelevant parameters [5,35,36,37,38]. It is common to have parameter directions $10^{-10}$ times as important as the most relevant one, but impossible to repeat an experiment the $M=10^{20}$ times needed to bridge this gap. Sometimes it is possible to remove the irrelevant parameters and derive a simpler effective theory. This is what happens in physics, where a large separation of scales allows great simplicity and high accuracy [39,40]. However, many other systems we would like to model cannot, or cannot yet, be so simplified. For complicated biological reactions, climate models, or neural networks, it is unclear which of the microscopic details can be safely ignored, or what the right effective variable is. Unlike our toy models, we cannot easily adjust *d*, since every parameter has a different meaning. This is why we seek statistical methods which do not require us to find the right effective theory. Furthermore, in particular, here we study priors almost invariant to complexity.

The optimal prior is discrete, which makes it difficult to find, and this difficulty appears to be why its good properties have been overlooked. It is known analytically only for extremely simple models such as M=1 Bernoulli, and previous numerical work only treated slightly more complicated models, with d≤2 parameters [9]. While our concern here is with the ideal properties, for practical use nearly optimal approximations may be required. One possibility is the adaptive slab-and-spike prior introduced in [6]. Another would be to use some variational family $p_{\lambda }\left(\theta \right)$ with adjustable meta-parameters λ [41].

Discreteness is also how the exactly optimal $p_{⋆}\left(\theta \right)$ encodes a length scale L≈1 in the model geometry, which is the divide between relevant and irrelevant parameters, between parameters that are constrained by data and those which are not. Making this distinction in some way is essential for good behavior, and it implies a dependence on the quantity of data. An effective model appropriate for much fewer data than observed will be too simple: the atoms of $p_{⋆}\left(\theta \right)$ will be too far apart (much like recording too few significant figures), or else selecting a small *d* means picking just one edge (fixing some parameters which may, in fact, be relevant). On the other hand, what we have demonstrated here is that a model appropriate for much more data—infinitely more in the case of $p_{J}\left(\theta \right)$—will instead introduce enormous bias into our inference about θ.

## Figures and Tables

**Figure A1:**
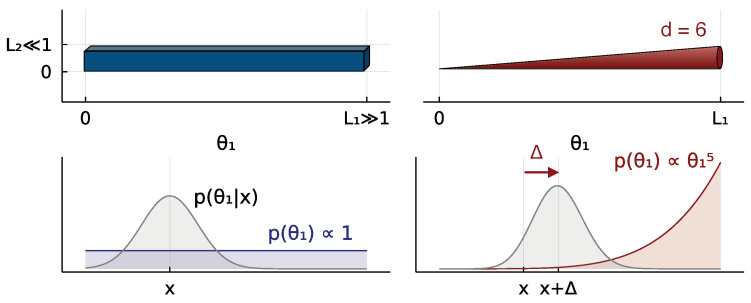
The simplest geometry in which to see measure-induced posterior bias. We compare two d=6 model manifolds, both with $\theta_{1}$ relevant, Fisher length $L_{1}\gg 1$, and five irrelevant dimensions ($L_{\mu }\ll 1$, μ=2,3,…6) which are either of constant size (**left**) or taper linearly (**right**). The distinction between these two situations is, by assumption, unobservable, but using the *d*-dimensional notion of volume as a prior gives an effective $p(\theta_{1})$ which is either flat or $\propto {\left(\theta_{1}\right)}^{5}$. This can induce substantial bias in the posterior $p(\theta_{1}|x)$.

## Data Availability

The code used to find the priors (and the scores) shown is available at https://github.com/mcabbott/AtomicPriors.jl, using Julia [42].

## Appendix A

For brevity, the main text omits some standard definitions. The KL divergence (or relative entropy) is defined

$$D_{\mathrm{KL}}\left[p(x)∥q(x)\right]=\int dx\;p\left(x\right)\;\log\frac{p(x)}{q(x)}.$$

With a conditional probability, in our notation $D_{\mathrm{KL}}\left[p(x|\theta )∥q(x)\right]$ integrates *x* but remains a function of θ. The Fisher information metric $g_{\mu \nu }\left(\theta \right)$ is the quadratic term from expanding $D_{\mathrm{KL}}\left[p(x|\theta +d\theta )∥p(x|\theta )\right]$.

The mutual information is

$$\begin{array}{cc} I(X;\Theta ) & =D_{\mathrm{KL}}\left[p(x,\theta )∥p(x)p(\theta )\right]\\ \; & =S(\Theta )-S(\Theta |X)\\ \; & =\;\int \int \;dx\;d\theta \;p\left(x|\theta \right)p\left(\theta \right)\log\frac{p(x|\theta )}{p(x)} \end{array}$$

where we use Bayes theorem,

p(θ|x)=p(x|θ)p(θ)/p(x)

with p(x)=∫dθp(x|θ)p(θ), and entropy

S(X)=−∫dxp(x)logp(x).

Conditional entropy is S(X|θ)=−∫dxp(x|θ)logp(x|θ) for one value θ, or S(X|Θ)=∫dθp(θ)S(X|θ). With a Gaussian likelihood, Equation (7), and $X=R^{m}$, this is a constant:

$$S\left(X|\Theta \right)=\frac{m}{2}\left(1+\log2\pi \sigma^{2}\right).$$

Many of these quantities depend on the choice of prior, such as the posterior p(θ|x) and the mutual information I(X;Θ). This is also true of our bias pressure b(θ) and worst-case $B=\max_{\theta }b\left(\theta \right)$. When we need to refer to those for a specific prior, such as $p_{⋆}\left(\theta \right)$, we use the same subscript, writing $I_{⋆}$ and $B_{⋆}.$

All probability distributions are normalized. In particular, the gradient in (5) is taken with the constraint of normalization—varying the density at each point independently would give a different constant.

### Appendix A.1. Square Hypercone

Here we consider an even simpler toy model, in which we can more rigorously define what we mean by co-volume, and analytically calculate the posterior deviation.

Consider a *d*-dimensional cone consisting of a line of length *L* thickened to have a square cross-section. This is $y\left(\theta \right)=\left(\theta_{1},r\theta_{2},r\theta_{3},\ldots \right)$ with scale $r\left(\theta_{1}\right)=\theta_{1}/L$, and co-ordinate ranges:

$$\begin{array}{cc} 0 & \leq \theta_{1}\leq L\\ 0 & \leq \theta_{\mu }\leq 1,\;\mu =2,3,\ldots ,d. \end{array}$$

Fixing noise σ=1, this has one relevant dimension, length *L*, and d−1 irrelevant dimensions whose lengths are always ≤1. The FIM is then

$$g\left(\theta \right)=\left[\begin{array}{cccc} 1+\sum_{\mu =2}^{d}r^{2} & \frac{\theta_{1}\theta_{2}}{L^{2}} & \frac{\theta_{1}\theta_{3}}{L^{2}} & \cdots \\ \frac{\theta_{1}\theta_{2}}{L^{2}} & r^{2} & 0\\ \frac{\theta_{1}\theta_{3}}{L^{2}} & 0 & r^{2}\\ \vdots & & & \ddots \end{array}\right].$$

Thus the volume element is

$$\sqrt{\det g(\theta )}=1\;r{\left(\theta_{1}\right)}^{\left(d-1\right)}.$$

Regarding the first factor as $\sqrt{\det g_{\mathrm{rel}}}+O\left(1/L^{2}\right)$, it is trivial to integrate the second factor over $\theta_{\mu }$ for all μ≥2, and this factor $\int_{0}^{1}d\theta_{2}\cdots \int_{0}^{1}d\theta_{d}\sqrt{\det g_{\mathrm{irrel}}}={\left(r\left(\theta_{1}\right)\right)}^{\left(d-1\right)}$ is the irrelevant co-volume. The effective Jeffreys prior along the one relevant dimension is thus

$$p_{J}\left(\theta_{1}\right)\propto {\left(\theta_{1}\right)}^{\left(d-1\right)}$$

which clearly has much more weight at large $\theta_{1}$, at the thick end of the cone.

Now observe some *x*, giving $p\left(\theta_{1}|x\right)\propto e^{-{\left(x-\theta_{1}\right)}^{2}/2}{\left(\theta_{1}\right)}^{\left(d-1\right)}$. With a few lines of algebra, we can derive, assuming 1≪x≪L, that the posterior deviation (9) is

$$\Delta =x-{\langle \theta_{1}\rangle }_{p(\theta_{1}|x)}=\frac{d-1}{x}+O\left(\frac{1}{x^{3}}\right).$$

Choosing L=50 and d=26 to roughly match Figure 3, at x≈10 the deviation is Δ≈2.5. This is smaller than what is seen for the exponential decay model, whose geometry is, of course, more complicated. This difference is also detected by bias pressure. The maximum b(θ) for this cone is about 55 bits, which is close to the d=5 model in Figure 5.

While this example takes all irrelevant dimensions to be of equal Fisher length, it would be more realistic to have a series $L,1,L^{-1},L^{-2},\ldots$ equally spaced on a log scale. This makes no difference to the effective $p_{J}\left(\theta_{1}\right)\propto {\left(\theta_{1}\right)}^{(d-1)/2}$ and hence to the posterior deviation Δ. Figure A1 draws instead a cone with a round cross-section, which also makes no difference. It compares this to a shape of constant cross-section, for which $p_{J}\left(\theta_{1}\right)\propto 1$; hence, there is no such bias.

### Appendix A.2. Estimating I(X;*Θ*) and Its Gradient

The mutual information can be estimated for a discrete prior

$$p\left(\theta \right)=\sum_{a=1}^{K}\lambda_{a}\delta \left(\theta -\theta_{a}\right)$$

and a Gaussian likelihood (7) by replacing the integral over *x* with normally distributed samples:

$$I\left(X;\Theta \right)=\sum_{a}\lambda_{a}\;\;\underset{\mathrm{sample}\;x∼N(\theta_{a},\sigma^{2})}{\underset{︸}{\int dx\;p(x|\theta_{a})}}\;\;\log\frac{p(x|\theta_{a})}{\sum_{b}\lambda_{b}p\left(x|\theta_{b}\right)}.$$

This gives an unbiased estimate, and what we used for plotting I(X;Θ) in Figure 2 and Figure 5.

However, for finding $p_{⋆}\left(\theta \right)$, what we need is the very small gradients of *I* with respect to each $\theta_{a}$: near to the optimum, the function is very close to flat. We find that, instead of Monte Carlo, the following kernel density approximation works well:

(A1) $$\begin{array}{cc} S(X) & =-\sum_{a}\lambda_{a}\int dx^{\prime }\;p\left(x^{\prime }|\theta_{a}\right)\log\sum_{b}\lambda_{b}\;p\left(x^{\prime }|\theta_{b}\right)\\ \; & =-\sum_{a}\lambda_{a}\int dx^{\prime }\;p\left(x^{\prime }|\theta_{a}\right)[\log\sum_{b}\lambda_{b}\;p\left(x=\theta_{a}|\theta_{b}\right)\\ \; & \;\;\;\;+O{\left(x^{\prime }-\theta_{a}\right)}^{2}]\\ \; & \approx -\sum_{a}\lambda_{a}\log\sum_{b}\lambda_{b}\;e^{{\left[y\left(\theta_{a}\right)-y\left(\theta_{b}\right)\right]}^{2}/2\sigma^{\prime 2}}+\mathrm{const}. \end{array}$$

Here we Taylor expand the logp(x) about $x=\theta_{a}$ for each atom [43]. For the purpose of finding $p_{⋆}\left(\theta \right)$ we may ignore the constant and the conditional entropy in I(X;Θ)=S(X)−S(X|Θ). Further, this is a better estimate used at $\sigma^{\prime }=\sqrt{2}\sigma$.

Before maximizing I(X;Θ) using L-BFGS [44,45] to adjust all $\theta_{a}$ and $\lambda_{a}$ together, we find it useful to sample initial points using Mitchell’s best-candidate algorithm [46].

### Appendix A.3. Other Methods

Our focus here is on the properties of the optimal prior $p_{⋆}\left(\theta \right)$, but better ways to find nearly optimal solutions may be needed in order to use these ideas on larger problems. Some ideas have been explored in the literature:

- The famous algorithm for finding $p_{⋆}\left(\theta \right)$ is due to Blahut and Arimoto [47,48], but it needs a discrete Θ, which limits *d*. This was adapted to use MCMC sampling instead by [49], although their work appears to need discrete *X* instead. Perhaps it can be generalized.
- More recently, the following lower bound for I(X;Θ) was used by [41] to find approximations to $p_{⋆}\left(\theta \right)$:
  $$I\left(X;\Theta \right)\geq I_{\mathrm{NS}}=-S\left(X|\Theta \right)-\int \;dx\;p\left(x\right)\log\max_{\theta }p\left(x|\theta \right).$$
  This bound is too crude to see the features of interest here: For all models in this paper, it favors a prior $p_{2}\left(\theta \right)={\mathrm{argmax}}_{p(\theta )}I_{\mathrm{NS}}$ with just two delta functions, for any noise level σ.
- We mentioned above that adjusting some “meta-parameters” of some distribution $p_{\bar{\theta },\bar{\sigma }}\left(\theta \right)$ would be one way to handle near-optimal priors. This is the approach of [41], and of many papers maximizing other scores, often described as “variational”.
- Another prior that typically has large I(X;Θ) was introduced in [6] under the name “adaptive slab-and-spike prior”. It pulls every point *x* in a distribution
  $$p_{\mathrm{NML}}\left(x\right)=\frac{\max_{\hat{\theta }}p\left(x|\hat{\theta }\right)}{Z},\;Z=\int \;dx\;\max_{\hat{\theta }}p\left(x|\hat{\theta }\right)$$
  back to its maximum likelihood point $\hat{\theta }$:
  $$p_{\mathrm{proj}}\left(\theta \right)=\int \;dx\;p_{\mathrm{NML}}\left(x\right)\;\delta \left(\theta -\underset{\hat{\theta }}{\mathrm{argmax}}p\left(x|\hat{\theta }\right)\right).$$
  The result has weight everywhere in the model manifold, but has extra weight on the edges. Because the amount of weight on edges is controlled by σ, it adopts an appropriate effective dimensionality (8), and has a low bias (5).

### Appendix A.4. Bias Alla Bennett

The KL divergence integral needed for b(θ) is somewhat badly behaved when the prior’s weight is far from point θ. To describe how we handle this, we begin with the naïve Monte Carlo calculation of p(x)=∫dθp(θ)p(x|θ), which involves sampling from the prior. If *x* is very far from where the prior has most of its weight, then we will never obtain any samples where p(x|θ) is not exponentially small, so we will miss the leading contribution to p(x).

Sampling from the posterior p(θ|x)∝p(θ)p(x|θ) instead, we will obtain points in the right area, but the wrong answer. The following identity due to Bennett [26] lets us walk from the prior to the posterior and obtain the right answer, sampling from distributions $\propto p\left(\theta \right)\;p{\left(x|\theta \right)}^{\alpha }$ for several powers α. Defining $\Delta_{\alpha }^{\delta }\left(x\right)$:

$$\begin{array}{cc} 0 & =\alpha_{0}<\alpha_{1}<\ldots <\alpha_{n}=1\\ e^{\Delta_{\alpha }^{\delta }\left(x\right)} & ={\langle p{\left(x|\theta \right)}^{\delta }\rangle }_{x,\alpha }=\frac{\int d\theta \;p\left(\theta \right)\;p{\left(x|\theta \right)}^{\alpha }\;p{\left(x|\theta \right)}^{\delta }}{\int d\theta \;p\left(\theta \right)\;p{\left(x|\theta \right)}^{\alpha }} \end{array}$$

the result is

$$\log p\left(x\right)=\sum_{i=0}^{n-1}\Delta_{\alpha_{i}}^{\left(\alpha_{i+1}-\alpha_{i}\right)}\left(x\right)=-\sum_{i=1}^{n}\Delta_{\alpha_{i}}^{\left(\alpha_{i-1}-\alpha_{i}\right)}\left(x\right)$$

i.e.,

p(x)=∏α≠1p(x|θ)αnext−α1x,α=1/∏α≠01/p(x|θ)α−αprev1x,α.

For n=1 the first is trivial, and the second reads $1/p\left(x\right)={\langle 1/p(x|\theta )\rangle }_{\theta ∼p(\theta |x)}$.

Next, we want $D_{\mathrm{KL}}\left[p\left(x|\varphi \right)∥p\left(x\right)\right]$ which involves −∫dxp(x|φ)logp(x). To plug this in, we would need to average ${\langle \ldots \rangle }_{x,\alpha }$ at every *x* in the integral. Rather than sample from a fresh set of α-distributions for each *x*, we can take one set at some $x_{0}$, and correct using importance sampling to write:

$${\langle \ldots \rangle }_{x,\alpha }={\langle \ldots \frac{p(x|\theta )}{p(x_{0}|\theta )}\rangle }_{x_{0},\alpha }$$

All of this can be performed without knowing the normalization of the prior, *Z* in (2).

The same set of samples from α-distributions can be used to calculate either forward or backward. These give upper and lower bounds on the true value. When they are sufficiently far apart to be visible, the plots show both of them. Figure 2 uses, as the α-distributions, all larger σ-values on the plot, and also shows (dotted line) the result of naïve sampling from $p_{J}\left(\theta \right)$. Figure 5 uses about 30 steps.

### Appendix A.5. Jeffreys and Vandermonde

To find Jeffreys prior, let us parameterize the exponential decay model (6) by $\phi_{\mu }=e^{-\exp(\theta_{\mu })}$ which lives in the unit interval:

$$y_{t}\left(\theta \right)=\sum_{\mu =1}^{d}\frac{e^{-k_{\mu }t}}{d}=\sum_{\mu =1}^{d}\frac{{\left(\phi_{\mu }\right)}^{t}}{d}$$

where $\theta_{\mu }=\log k_{\mu }\in R,\;0\leq \phi_{\mu }\leq 1$. The Fisher information metric (1) reads $g_{\mu \nu }=\frac{1}{\sigma^{2}}\sum_{t}^{m}J_{\mu t}J_{\nu t}$ in terms of a Jacobian which, in these co-ordinates, takes the simple form:

$$J_{\mu t}\left(\phi \right)=\frac{\partial y_{t}}{\partial \phi_{\mu }}=\frac{1}{d}t\;\phi^{t-1}.$$

In high dimensionality, $g_{\mu \nu }$ is very badly conditioned, and thus it is difficult to find the determinant with sufficient numerical accuracy (or at least, it is slow, as high-precision numbers are required). However, in the case d=m, where the Jacobian is a square matrix, it is of Vandermonde form. Hence, its determinant is known exactly, and we can simply write:

$$\begin{array}{cc} p_{J}\left(\phi \right) & =\sqrt{\det g_{\mu \nu }\left(\phi \right)}=\left|\det J_{\mu t}\left(\phi \right)\right|\\ \; & =\prod_{1\leq \mu <\nu \leq d}\left|\phi_{\mu }-\phi_{\nu }\right|\prod_{t=1}^{m}\frac{t}{d}. \end{array}$$

For d≠m, more complicated formulae (involving a sum over Schur polynomials) are known, but the points in Figure 5 are chosen not to need them.

To sample from Jeffreys prior, or posterior, we use the affine-invariant “emcee” sampler [50]. This adapts well to badly conditioned geometries. Because the Vandermonde formula lets us work in machine precision, we can sample $10^{6}$ points in a few minutes, which is sufficient for Figure 3.

In the enzyme kinetics models of Figure 6, finding $y_{t}\left(\theta \right)$ involves solving a differential equation. The gradient $\partial y_{t}/\partial \theta_{\mu }$ is needed both for Jeffreys density and for maximizing S(X) via the above KDE Formula (A1). This can be handled efficiently by passing dual numbers through the solver [51].

### Appendix A.6. Michaelis–Menten et al.

The arrows in (11) summarise the following differential equations for the concentrations of the four chemicals involved:

$$\begin{array}{cc} \partial_{t}\left[E\right] & =-k_{f}\left[E\right]\left[S\right]+k_{r}\left[ES\right]+k_{p}\left[ES\right]\\ \partial_{t}\left[S\right] & =-k_{f}\left[E\right]\left[S\right]+k_{r}\left[ES\right]\\ \partial_{t}\left[ES\right] & =+k_{f}\left[E\right]\left[S\right]-k_{r}\left[ES\right]-k_{p}\left[ES\right]\\ \partial_{t}\left[P\right] & =k_{p}\left[ES\right]. \end{array}$$

These equations conserve $E_{0}=\left[E\right]+\left[ES\right]$ (the enzyme is recycled) and $S_{0}=\left[S\right]+\left[ES\right]+\left[P\right]$ (the substrate is converted to product), leaving two dynamical quantities, [S] and [P]. The plot takes them to have initial values [S]=1, [P]=0 at t=0, and we fix $E_{0}=1/4$, $S_{0}=1$.

The original analysis of Michaelis and Menten [52] takes the first two reactions to be in equilibrium. This can be viewed as taking the limit $k_{r},k_{f}\to \infty$ holding fixed $K_{D}=k_{r}/k_{f}$, which picks a 2-parameter subspace of Θ, an edge of the manifold. Then, $\left[ES\right]=\left[E\right]\left[S\right]/K_{D}$ becomes constant, leaving their equation

(A2) $$\partial_{t}\left[P\right]=\frac{k_{p}E_{0}\left[S\right]}{K_{D}+\left[S\right]}.$$

If we do not observe [E], then this is almost identical to the quasi-static limit of Briggs and Haldane [53], who take $k_{r},E_{0}\to 0$, and $k_{f},k_{p}\to \infty$ holding fixed $K_{M}=k_{p}/f_{f}$ and $V_{\max}=k_{p}E_{0}$, which gives

$$\partial_{t}\left[P\right]=\frac{V_{\max}\left[S\right]}{K_{M}+\left[S\right]}$$

which was much later shown to be analytically tractable [54].

In Figure 6, most of the points of weight in the optimal prior lie on the intersection of these two 2-parameter models, that is, on a pair of one-parameter models. These and other limits were discussed geometrically in [28].

For the d=8 ping-pong model (12), we take initial conditions of [A]=[B]=1, [E]=[E∗]=0.5, [EA]=[E∗P]=[E∗B]=[EQ]=0.1, [P]=[Q]=0.

### Appendix A.7. Ever since the Big Bang

The claim that the age of the universe constrains us from taking the asymptotic limit in realistic multi-parameter models deserves a brief calculation. The conventional age is $13.8\times 10^{8}$ years, about $4.4\times 10^{17}$ s [55].

For the model (6), manifold widths are shown to scale such as $L\propto \sqrt{\mathrm{eig}g}$ in [23], and [5,23] shows FIM eigenvalues $\propto 10^{-d}$. Repeating an experiment *M* times scales lengths by $\sqrt{M}$, so we need roughly $10^{d}$ repetitions to make the smallest manifold width larger than 1. If the initial experiment took 1 s, then it is impossible to perform enough repetitions to make all dimensions of the d=26 model relevant.

Other models studied in [5,23] have different slopes log(eigg) vs. *d*, so the precise cutoff will vary. However, models with hundreds, or thousands, of parameters are also routine. It seems safe to claim that for most of these, the asymptotic limit is never justified.

### Appendix A.8. Terminology

What we call Jeffreys prior, $p_{J}\left(\theta \right)$, is (apart from variations in apostrophes and articles) sometimes called “Jeffreys’s nonlocation rule” [56] in order to stress that it is not quite what Jeffreys favored. He argued for separating “location” parameters (such as an unconstrained position ϕ∈R) and excluding them from the determinant. The models we consider here have no such perfect symmetries.

What we call the optimal prior $p_{⋆}\left(\theta \right)$ is sometimes called “Shannon optimal” [17], and sometimes called a “reference prior” after Bernardo [8]. The latter is misleading, as definition 1 in [8] explicitly takes the asymptotic limit M→∞. This is then Jeffreys prior, until some ways to handle nuisance parameters are appended. The idea of considering ${\mathrm{argmax}}_{p(\theta )}I\left(X;\Theta \right)$ in the limit is older, for instance Lindley [7] considers it, and also notes that it leads to Jeffreys prior (which he does not like for multi-parameter models, but not for our reasons). In [15], the discrete prior for *k* repetitions is called the “*k*-th reference prior”, and is understood to be discrete, but is of interest only as a tool for showing that the limit exists. We stress that the asymptotic limit removes what are (for this paper) the interesting features of this prior.

The same $p_{⋆}\left(\theta \right)$ can also be obtained by an equivalent minimax game, for which Kashyap uses the term “optimal prior” [10]. However, he, too, takes the asymptotic limit.
